# Examining the potential public health benefit of offering STI testing to men in amateur football clubs: evidence from cross-sectional surveys

**DOI:** 10.1186/s12889-015-1951-7

**Published:** 2015-07-17

**Authors:** Catherine H. Mercer, Sebastian S. Fuller, John M. Saunders, Pamela Muniina, Andrew J. Copas, Graham J. Hart, Lorna J. Sutcliffe, Anne M. Johnson, Jackie A. Cassell, Claudia S. Estcourt

**Affiliations:** Research Department of Infection and Population Health, University College London, London, UK; Centre for Immunology and Infectious Disease, Blizard Institute of Cell & Molecular Science, Barts and The London School of Medicine and Dentistry, London, UK; Division of Primary Care and Public Health, Brighton and Sussex Medical School, University of Sussex, Brighton, UK

**Keywords:** Sexually transmitted infections, Sexual health, Public health, Sport, Men

## Abstract

**Background:**

In Britain, young people continue to bear the burden of sexually transmitted infections (STIs) so efforts are required, especially among men, to encourage STI testing. The SPORTSMART study trialled an intervention that sought to achieve this by offering chlamydia and gonorrhoea test-kits to men attending amateur football clubs between October and December 2012. With football the highest participation team sport among men in England, this paper examines the potential public health benefit of offering STI testing to men in this setting by assessing their sociodemographic characteristics, sexual behaviours, and healthcare behaviour and comparing them to men in the general population.

**Methods:**

Data were collected from 192 (male) members of 6 football clubs in London, United Kingdom, aged 18–44 years via a 20-item pen-and-paper self-completion questionnaire administered 2 weeks after the intervention. These were compared to data collected from 409 men of a similar age who were resident in London when interviewed during 2010–2012 for the third National Survey of Sexual Attitudes and Lifestyles (Natsal-3), a national probability survey that used computer-assisted-personal-interviewing with computer-assisted-self-interview. Age standardisation and multivariable regression were used to account for sociodemographic differences between the surveys.

**Results:**

Relative to men in the general population, SPORTSMART men were younger (32.8 % *vs.* 21.7 % aged under 25 y), and more likely to report (all past year) at least 2 sexual partners (adjusted odds ratio, AOR: 3.25, 95 % CI: 2.15–4.92), concurrent partners (AOR: 2.05, 95 % CI: 1.39–3.02), and non-use of condoms (AOR: 2.17, 95 % CI: 1.39–3.41). No difference was observed in STI/HIV risk perception (AOR for reporting “not at all at risk” of STIs: 1.25, 95 % CI: 0.76–2.04; of HIV: AOR: 1.54, 95 % CI: 0.93–2.55), nor in reporting STI testing in the past year (AOR: 0.83, 95 % CI: 0.44–1.54), which was reported by only one in six men.

**Conclusions:**

Relative to young men in the general population, football club members who completed the SPORTSMART survey reported greater sexual risk behaviour but similar STI/HIV risk perception and STI testing history. Offering STI testing in amateur football clubs may therefore widen access to STI testing and health promotion messages for men at higher STI risk, which, given the minority currently testing and the popularity of football in England, should yield both individual and public health benefit.

## Background

In Britain, young people continue to bear the burden of sexually transmitted infections (STI) [1], despite the introduction of a number of strategies in recent years to improve their access to sexual healthcare [2–7]. It is widely recognised that greater effort is required to get men to test for STIs, including through increasing their engagement with screening [8]. Although healthcare settings may be preferred [9], previous research suggests that young men are open to STI testing in a range of settings, including non-healthcare settings, such as in sports venues [9–11]. As such, we developed, piloted, and evaluated two feasible, replicable interventions to promote and deliver STI screening targeting young men in amateur football clubs – ‘SPORTSMART’ [12]. The hypothesis being that this approach to STI screening may reach men who would not otherwise use sexual health services and could provide a generalisable solution for other health promotion interventions in young men such as drug, smoking, and alcohol awareness. Uptake of the SPORTSMART intervention was high at almost 60 % implying high acceptability of screening in this setting [12]. This paper examines the potential public health benefit of offering STI testing to men in this setting by assessing club members’ sociodemographic characteristics, sexual behaviours, and sexual healthcare behaviour and comparing them to men in the general population.

## Methods

### The SPORTSMART survey

The development and evaluation of the SPORTSMART intervention has been reported elsewhere [12]. Briefly, we developed two interventions to explore the acceptability and feasibility of urine-based STI (chlamydia and gonorrhoea) screening interventions targeting men in amateur football clubs in London, United Kingdom. Between October and December 2012, we tested these interventions in a pilot cluster randomised control trial involving six amateur football clubs. Two weeks after the intervention was completed, all club members aged at least 18 years were invited to take part in a brief, self-completion, anonymous, pen-and-paper questionnaire to assess their sexual risk behaviour and STI testing history, as well as collect standard sociodemographic data. Club managers, secretaries, and team captains offered the survey to all male members aged at least 18 years present at the time, and completed surveys were returned to a locked box on display in the club premises. As far as possible, questions used the same wording as in the third National Survey of Sexual Attitudes and Lifestyles (Natsal-3) [13], to permit comparisons with this general population survey.

### The Natsal-3 survey

Full details of the methods of Natsal-3 have been reported elsewhere [13, 14]. Briefly, Natsal-3 is a stratified probability sample survey of 15,162 men and women aged 16–74 years in Britain who were interviewed between September 2010 and August 2012 (6,293 men). Participants were interviewed with a combination of computer-assisted face-to-face and self-completion questionnaires, which included questions about participants’ sexual lifestyles and attitudes. To ensure the greatest comparability with the SPORTSMART sample, only men in the Natsal-3 sample aged 18–44 years and resident in London were included in these analyses.

### Statistical analysis

Data from the two surveys were compared using the survey analysis functions of the statistical software Stata, version 13, enabling us to account for the weighting, clustering and stratification of the Natsal-3 data. Survey weights were applied to the Natsal-3 data to adjust for unequal probability of selection and non-response, ensuring these data were broadly representative of the British general population, according to the 2011 Census, in terms of gender, age group and region [13, 14].

As significant differences were observed in the age profile of men in the two surveys, age standardisation was used to calculate age-adjusted prevalence estimates for all the sociodemographic characteristics, sexual behaviours, and sexual health outcomes for which comparable data are available for the two surveys. Multivariable logistic regression was then used to calculate adjusted odds ratios (AORs) to account for the confounding effect of differences in age and educational attainment between the two samples. Multivariable logistic regression was also used to calculate AORs to take account of differences between the two samples in the number of sexual partners reported (in addition to age and educational attainment) when comparing the data on non-use of condoms, STI/HIV risk perception, and sexual health outcomes. Statistical significance is considered as *p* < 0.05 for all analyses.

Data are also reported for the type of setting where SPORTSMART men tested for any STI in the past year, (excluding testing as part of the SPORTSMART intervention). The corresponding data for Natsal-3 refer specifically to testing for chlamydia in the past year. Formal statistical comparisons are not made due to small numbers.

### Ethics

Ethical approval for the SPORTSMART study was given by the National Research Ethics Service (reference 13/SC/0029). The Natsal-3 study obtained ethics approval from Oxfordshire Research Ethics Committee A (reference 09/H0604/27).

## Results

In total, 192 men aged 18–44 years completed the SPORTSMART survey. This corresponds to an estimated response rate of 61.9 % of the total number of questionnaires left with the football clubs. In the Natsal-3 sample, 409 men were in this age-range and resident in London. The estimated overall response rate for Natsal-3 was 57.7 % and the co-operation rate was 65.8 % (the number of interviews completed at eligible addresses for which contact was made) [13, 14].

### Sociodemographic characteristics

Men who completed the SPORTSMART survey were younger than London-resident men in the Natsal-3 sample, with 32.8 % aged under 25 years in contrast to 21.7 % (Table 1). After taking this age difference into account, the SPORTSMART men were still more likely to report a university education (65.1 % *vs.* 52.2 %, AOR: 1.83), and current regular sexual partners (76.5 % *vs.* 60.6 %, AOR: 1.92), but were slightly less likely to live with a partner (47.8 % *vs.* 53.8 %, AOR: 0.64).

**Table 1:** Sociodemographic and behavioural profiles of the SPORTSMART sample compared to the general population^a^

Sample:	General population^a^	SPORTSMART		
Denominator	623, 409^b^	192	Odds ratio^c^ (95 % CI) adjusted for:	
	Age-adjusted % (95 % CI)	Age-adjusted % (95 % CI)	Age & educational attainment^d^	Age, educational attainment^d^ & partner numbers
Sociodemographics				
Aged under 25 years	21.7 %^e^ (17.8–26.0 %)	32.8 %^e^ (26.5–39.8 %)	-	-
Educational attainment: Has a degree (or higher) qualification	52.2 % (46.3–58.1 %)	65.1 % (60.0–70.3 %)	1.83 (1.26–2.65) *p* = 0.002	-
Relationship status: Cohabiting with a partner	53.8 % (48.9–58.8 %)	47.8 % (42.1–53.5 %)	0.64 (0.40–1.01) *p* = 0.057	
Currently has regular sexual partner(s)	60.6 % (55.3–65.9 %)	76.5 % (71.6–81.3 %)	1.92 (1.25–2.97) *p* = 0.003	-
Sexual partners, past year				
At least 1 partner	90.0 % (87.5–92.5 %)	97.0 % (94.6–99.4 %)	4.51 (1.84–11.1) *p* = 0.001	-
At least 2 partners	20.0 % (16.3–23.8 %)	40.2 % (33.6–46.8 %)	3.25 (2.15–4.92) *p* < 0.001	-
Concurrent partners	7.6 % (5.0–10.1 %)	17.2 % (12.6–21.8)	2.05 (1.39–3.02) *p* < 0.001	0.93 (0.50–1.76) *p* = 0.829
At least 1 same-sex partner	3.6 % (1.7–5.4 %)	3.7 % (1.1–6.3 %)	1.30 (0.53–3.20) *p* = 0.564	0.63 (0.16–2.42) *p* = 0.499
Paid for sex	2.1 % (<0.1–4.2 %)	2.8 % (0.1–4.9 %)	3.33 (1.04–10.7) *p* = 0.043	2.53 (0.75–8.60) *p* = 0.136
Non-use of condoms, past year				
Non-use of condoms on at least one occasion	70.0 % (64.6–75.6 %)	81.4 % (76.0–86.9 %)	2.17 (1.39–3.41) *p* = 0.001	1.57 (0.93–2.64) *p* = 0.089
At least 2 partners & no condom use during this time	4.2 % (2.2–6.2 %)	5.0 % (2.2–7.7 %)	2.10 (1.02–4.33) *p* = 0.044	0.92 (0.36–2.40) *p* = 0.879
STI/HIV risk perception				
“Not at all at risk” of STIs	57.5 % (51.8–63.3 %)	51.9 % (45.6–58.1 %)	0.79 (0.54–1.15) *p* = 0.224	1.25 (0.76–2.04) *p* = 0.382
“Not at all at risk” of HIV	62.5 % (57.1–67.9 %)	58.8 % (52.8–64.9 %)	0.93 (0.63–1.37) *p* = 0.726	1.54 (0.93–2.55) *p* = 0.094
Sexual health outcomes				
STI testing, past year^f,g^	15.5 % (12.0–19.0 %)	16.7 % (11.6–21.7 %)	1.25 (0.75–2.06) *p* = 0.389	0.83 (0.44–1.54) *p* = 0.547
STI diagnosis/es, ever	13.9 % (9.8–17.9 %)	16.3 % (11.3–21.3 %)	1.07 (0.61–1.86) *p* = 0.815	0.69 (0.35–1.34) *p* = 0.272

^a^General population defined as men aged 18–44 years resident in London who participated in Natsal-3

^b^Weighted, unweighted denominators for Natsal-3; unweighted for SPORTSMART study

^c^Reference category is the general population sample

^d^In the multivariable analyses, educational attainment is categorised as reporting having a degree or postgraduate qualification *vs.* else

^e^Unadjusted percentages. Corresponding *p*-value: 0.004

^f^Where the SPORTSMART survey asked about their experience of STI testing, the question specified: “Do not include testing with SPORTSMART at your football club”

^g^The question in Natsal-3 corresponds just to testing for Chlamydia

### Sexual behaviour

Almost all men in the SPORTSMART sample (97.0 %) reported at least one sexual partner in the past year in contrast to 90.0 % of men in the Natsal-3 sample (AOR: 4.51), and a significantly larger proportion of men in the SPORTSMART sample reported two or more partners in the past year (40.2 % *vs.* 20.0 %; AOR: 3.25). Men in the SPORTSMART sample were also more likely to report having had concurrent sexual partners in the past year (17.2 % *vs.* 7.6 %, AOR: 2.05), but not when comparing just those men reporting two or more partners in this timeframe.

The age-adjusted prevalence estimates for reporting same-sex partner(s) in the past year were similar in the two surveys, at around 4 %, as were the estimates for having paid for sex in the past year.

Men in the SPORTSMART sample were more likely to report not using condoms for vaginal or anal sex on at least one occasion in the past year than men in Natsal-3 (81.4 % *vs.* 70.0 %), although not in the multivariable model after additionally adjusting for partner numbers (AOR: 1.57, 95 % CI: 0.93–2.64). A similar pattern was observed for the study’s measure of unsafe sex, defined as reporting at least two partners in the past year and no condom use during this time (AOR: 0.92, 95 % CI: 0.36–2.40).

### STI/HIV risk perception

The age-adjusted proportions of men who considered themselves to be “not at all at risk of STIs” were similar at just over half of men in each sample, as were the proportions who considered they were “not at all at risk of HIV” at around 60 % of men.

### Sexual health outcomes

Following age-standardisation, similar proportions reported having tested for at least one STI in the past year at around one in six men in the two samples. In both samples, STI testing was more common among men reporting at least two partners in the past year (as a marker of an individual’s STI risk behaviour) or who perceived themselves as at some risk of STIs (as a marker of their own and/or their partner’s risk behaviour) at 23.6 % (18.7–28.4 %). The age-adjusted proportions reporting having ever had STI diagnosis/es were also similar at around 14 %. In multivariable regression analyses, no significant differences were observed for either sexual health outcome.

### Venue of STI testing in the past year

Among the 37 men in the SPORTSMART sample who reported STI testing in the past year (not including testing via SPORTSMART) and who reported the venue of their most recent test (if more than one), 22 (59.5 %) reported that this was at a sexual health clinic. Seven (18.9 %) reported general practice, and a further three reported another type of clinic, such that the overwhelming majority (86.5 %) had tested in a healthcare setting. The remaining five men reported “a test you sent for from the internet” (two men) or “other” (three men). A similar distribution was observed for the 68 men in Natsal-3 who reported testing specifically for Chlamydia in the past year: the most commonly cited setting was a sexual health clinic (44.1 %), followed by general practice (17.7 %), with altogether 79.4 % citing testing in a healthcare setting.

## Discussion

Relative to men of an equivalent age in the general population, men attending amateur football clubs who completed the SPORTSMART survey were more likely to report a larger number of partners as well as concurrent partners. However, this higher sexual risk behaviour did not translate into greater STI/HIV risk perception or a greater likelihood of STI testing in the past year. Indeed, while STI testing was more common among men reporting these STI risk behaviours, around three-quarters of these men reported that they had not tested in the past year. Among the minority of men who had tested in the past year, most did so in a healthcare setting. Initiatives that offer STI testing in amateur football clubs, like the SPORTSMART intervention [12], may therefore help to widen access to STI testing and health promotion messages for men who are at higher STI/HIV risk. Since more men in England participate in football than any other team sport [15], with around half of men aged 18–35 years playing football at least once a month although not necessarily as part of a football club [9], and given the achieved uptake of STI testing observed via the SPORTSMART intervention in this setting [9, 12], these results are encouraging for both individual and public health improvement. Furthermore, the football clubs that participated in the SPORTSMART study were all situated in London, which as a large city in the UK offers many opportunities to access free sexual healthcare. It is highly likely that initiatives like SPORTSMART may yield greater public health benefit in more geographically-remote settings with more limited access to sexual healthcare [11].

It is important to consider whether methodological differences between SPORTSMART and Natsal-3 may account for some of the findings observed. For example, the SPORTSMART sample data may be subject to greater reporting bias as, although they were collected using an anonymous self-completion survey, the men participated alongside their peers. The direction of this bias is unclear as over- and under-reporting of behaviours are both plausible. It is also necessary to question the impact that participation in the SPORTSMART intervention may have had on participants’ responses in the subsequent survey. STI screening promotion featured in all three arms of the SPORTSMART intervention [12] so social desirability bias is probable. We conducted the survey after implementing the intervention to ensure that men who took up the offer of completing the SPORTSMART survey felt confident that they did so anonymously. This may not have been the case if we had included the survey at the same time as the intervention as this included personal-identifying information passed to the healthcare team. In addition, people may have different reasons for participating in a survey in contrast to an intervention. As the main outcome of interest of the parent study was uptake of the SPORTSMART intervention, we felt it was essential to ensure that uptake was comparable to what it would be if this were scaled-up and rolled-out to all football clubs; this could not have been done had we implemented the survey at the same time as the intervention or beforehand. Due to the anonymous nature of the survey, it is not possible to know which men who completed the survey also participated in the SPORTSMART intervention, so it is not possible to consider whether the uptake of testing via SPORTSMART varied according to risk behaviour and/or STI testing history.

The SPORTSMART survey was designed as a brief self-completion questionnaire and so captured data on a relatively small number of variables, and did not collect data on a number of variables that may influence access to healthcare and thus the likelihood of STI testing, including whether or not men were registered with a GP. Furthermore the question on ethnicity had very high item non-response in the SPORTSMART survey (>50 %), for reasons which are unclear, such that these data were excluded from the analyses. However, the questions used were identically-worded questions to those used in Natsal-3 for almost all the characteristics and behaviours compared, permitting valid comparisons. One notable exception is the question regarding STI testing. The SPORTSMART survey asked about testing for any STI, but Natsal-3 asked about testing for particular STIs, including chlamydia, as used in this analysis. While this may therefore explain some of the difference observed, this is perhaps a reasonable comparison as a chlamydia test is the minimum test to be offered when testing for STIs, regardless of setting [2, 6].

To ensure valid comparisons in terms of the men being compared it was necessary to limit the Natsal-3 denominator to men resident in London. Thus, although Natsal-3 has a large sample size overall, the number of participants eligible for this study may have limited our ability to detect statistically signficant differences, especially given our use of age standardisation and multivariable regression. However, it was necessary to use these statistical techniques to take account of the confounding effects of demographic and behavioural differences between the two samples. Furthermore, while Natsal-3 is broadly representative of the British general population [13], with a response rate in line with other major social surveys completed in Britain around the same time [16, 17], it is worth noting that response was slightly lower among young men resident in London, the population group of interest for this paper [14].

## Conclusion

In conclusion, men who completed the SPORTSMART survey were more likely to report key sexual risk behaviours in the past year but were no more likely to have tested for STIs during this time. Offering STI testing in amateur football clubs - regardless of mode of delivery [12] appears to be an acceptable way of reaching men at risk of STIs who do not perceive themselves at risk and who would not seek testing through traditional services. As well as increasing access, this approach may also help to normalise STI testing, provide opportunities for health promotion, and possibly engage men with sexual health issues more broadly. With the recent changes in sexual health service delivery and commissioning in England [5, 6], including the recognition of STI prevention as just one component of sexual health and well-being [18], and in turn, sexual health just one component of public health, this study provides empirical evidence to inform the planning and delivery of services and health promotion interventions to maximise public health benefit.
